# *Haemoproteus iwa* in Great Frigatebirds (*Fregata minor*) in the Islands of the Western Indian Ocean

**DOI:** 10.1371/journal.pone.0097185

**Published:** 2014-05-08

**Authors:** Matthieu Bastien, Audrey Jaeger, Matthieu Le Corre, Pablo Tortosa, Camille Lebarbenchon

**Affiliations:** 1 Centre de Recherche et de Veille sur les maladies émergentes dans l’Océan Indien, Sainte Clotilde, Reunion Island; 2 Laboratoire d’Ecologie Marine, Université de La Réunion, Saint Denis, Reunion Island; 3 Université de la Réunion, Saint Denis, Reunion Island; East China Normal University, China

## Abstract

Blood parasites of the sub-genus *Haemoproteus* have been reported in seabirds, in particular in species in the Suliformes order. These parasites are transmitted by hippoboscid flies of the genus *Olfersia*; strong specificity has been suggested between the vector and its vertebrate host. We investigated the prevalence of *Haemoproteus* infection in Suliformes and hippoboscid flies in two oceanic islands of the Western Indian Ocean: Europa and Tromelin. In total, 209 blood samples were collected from great frigatebirds (*Fregata minor*), masked boobies (*Sula dactylatra*) and red-footed boobies (*Sula sula*). Forty-one hippoboscid flies were also collected from birds. Seventeen frigatebirds and one fly collected on Europa tested positive for the presence of *Haemoproteus* parasites by polymerase chain reaction. Phylogenetic analyses based on partial sequences of the Cytochrome *b* gene showed that parasites were closely related to *Haemoproteus iwa* reported from frigatebirds in the Pacific Ocean and in the Caribbean. *Plasmodium* was also detected in a frigatebird on Europa; however, its placement on the phylogenetic tree could not be resolved. We provide strong support for transmission of blood parasites in seabirds in the Western Indian Ocean and suggest that migrations between the Pacific and the Indian oceans could favor the large-scale distribution of *Haemoproteus iwa* in frigatebird populations.

## Introduction

Animal migrations have the potential to enhance the global spread of pathogens and facilitate cross-species transmission [1]. Infectious diseases associated with wild birds have been particularly studied as these hosts can disperse infectious agents over long distances (*e.g.* avian influenza virus, West-Nile virus, *Borrelia burgdorferi*, or *Plasmodium* parasites); however, current knowledge on the role of seabirds in the transmission and dispersal of infectious agents remains limited.

Recent studies have shown that seabirds can be infected with blood parasites of the sub-genus *Haemoproteus* and *Parahaemoproteus* (genus *Haemoproteus*) [2]–[3]. *Haemoproteus* (referring to hereafter as the sub-genus) are commonly found in birds worldwide, with prevalence of infected hosts varying between species and geographic locations [2]–[3]. The transmission cycle of these blood parasites involve hippoboscid flies (Hippoboscidae), where they reach an infective stage (sporozoite) in the salivary glands. Hippoboscid flies are well adapted to live in bird feathers because of their dorso-ventrally flattened morphology. Despite the fully developed and functional wings, these flies usually remain nearby their host.

Historically, *Haemoproteus* parasites have been mainly reported in Columbiformes (*e.g.* doves), and recent studies have shown that they also can infect seabirds [2], [4]–[7]. Indeed, they have been reported in birds in the order Suliformes, in particular in frigatebirds. In these hosts, two species have been documented: *Haemoproteus iwa* (sub-genus *Haemoproteus*), commonly found in the Pacific (Tern Island, Galapagos Islands, Laysan Island and Isabel Island), Atlantic (Cayman Islands and Ascension Island) and Indian oceans (Christmas Island) [2], [5]; and *Haemoproteus valkiunasi* (sub-genus *Parahaemoproteus*), recently described in the Eastern Indian Ocean [7].

Great frigatebirds (*Fregata minor*), masked boobies (*Sula dactylatra)* and red-footed boobies *(Sula sula*) are the most abundant species of Suliformes in the Western Indian Ocean. Although an undetermined *Haemoproteus* species was previously documented in great frigatebirds in Aldabra (Seychelles) [8], the geographic distribution, hosts, vectors as well as prevalence in seabird populations remain to be assessed. Host specificity of the hippoboscid flies has also been suggested; however, information on vector ecology and population genetics remain limited in the islands of the Western Indian Ocean.

In this study, we investigated the prevalence and identity of Haemosporidian parasites in great frigatebirds, masked boobies and red-footed boobies in two isolated oceanic islands of the Western Indian Ocean: Europa (22°20′S, 40°22′E) and Tromelin (15°53′S, 54°31′E). Europa (28 km^2^) is a coralline island in the southern Mozambique Channel. It holds one of the most diverse and abundant seabird community of the Western Indian Ocean, with eight breeding species and more than a million pair of breeding seabirds. Red-footed boobies (3000 pairs) and great frigatebirds (1100 pairs) breed in mixed colonies in the dry *euphorbia* forest of the island [9]. Tromelin is a small coralline islet located 600 km North of Reunion Island. The seabird community has been greatly impacted by human pressure and introduced predators. To date, it holds only two species of breeding seabirds: masked boobies (800 pairs) and red-footed boobies (600 pairs) [10].

Molecular detection was performed on blood samples collected from great frigatebirds, masked boobies and red-footed boobies as well as on hippoboscid flies. For positive samples, partial Cytochrome *b* gene sequencing and phylogenetic analyses were carried out in order to identify parasite species. Hippoboscid flies belonging to the genus *Olfersia* are typically found infecting frigatebirds and boobies with evidence of strong host specificity [11]. In this study, we also provide molecular data for hippoboscid flies present on Europa and Tromelin.

## Materials and Methods

Samples collection (Table 1) was performed under the approval of the French Southern and Antarctic Lands (“Terres Australes et Antartiques Françaises”), administrator of Europa and Tromelin islands. Blood samples were collected from the brachial vein and stored in Longmire lysis buffer [12]. For samples collected on Europa in 2011, however, samples were stored in RNA NOW (BIOGENTEX, Seabrook, Texas, USA) since the primary purpose of sample collection was the detection of RNA viruses (Lebarbenchon et al. unpublished data). RNA extraction was performed with the QIAamp RNA Mini Kit (Qiagen, Hilden, Germany), except for samples conserved in RNA NOW that were extracted following the RNA NOW isolation and purification protocol. Samples were eluted in a final volume of 40 µL and reverse-transcription was performed on 20 µL of RNA product using a previously published protocol [13]. Hippoboscid flies were collected during bird handling for blood collection and stored individually in 70% ethanol. Because of the presence of sporozoites in the salivary glands (located in the thorax) and in order to limit detection of parasites in the digestive system, thoraxes were carefully separated and DNA was extracted using the DNEasy Blood and Tissue DNA extraction kit (Qiagen, Hilden, Germany) and eluted in a final volume of 40 µL.

**Table 1:** Origin and number of positive/collected samples for the presence of *Haemoproteus* and *Plasmodium* parasites.

Island	Host species	Collection date	Blood samples		Hippoboscid flies
			Adults	Chicks	
Europa	Great frigatebird (*Fregata minor*)	Dec. 2011	NS	9/18	NS
		Nov. 2012	9/20	3/10	1/11
	Red-footed booby (*Sula sula*)	Dec. 2011	0/30	NS	NS
Tromelin	Masked booby (*Sula dactylatra*)	Sep. 2012	0/30	0/40	0/15
	Red-footed booby (*Sula sula*)	Sep. 2012	0/32	0/29	0/15

NS: Not Sampled.

Semi-nested polymerase chain reactions (PCR) were used to detect the presence of *Haemoproteus* and *Plasmodium* genera in blood and fly samples [14]. Specifically, PCR targeting the mitochondrial Cytochrome *b* gene (479 bp) were performed with the HAEMNF1 and HAEMNR3 primers followed by an internal PCR with the HAEMNF1 and HAEMR2 primers as previously described [14]. The Go Taq Hot Start Green Master Mix (Promega Corporation, Madison, USA) was used in a final volume of 25 µL containing 5 µL of cDNA for the first amplification and 1 µL of the primary PCR for the second amplification. PCR conditions were used as in Hellgren *et al.* [14], except the number of cycles that was increased to 30 and 40 for the primary and secondary PCR, respectively. PCR were carried out in a BIO-RAD CFX96 Touch PCR system (BIO-RAD, Hercules, California, USA). Finally, partial Cytochrome Oxidase I encoding gene (698 bp) was amplified and sequenced to determine the putative species of nine Hippoboscid flies, as described in Folmer et al. [15]. PCR were carried out in a BIO-RAD CFX96 Touch PCR system. All amplification products were analyzed by electrophoresis on a 2% agarose gel stained with 0.4% GelRed (Biotum, Hayward, California, USA) and sequenced by Genoscreen (Lille, France).

Partial Cytochrome *b* (for parasites) and Cytochrome Oxidase I (for Hippoboscid flies) gene sequences were aligned using CLC 6.0.1. (CLC bio, Aarhus, Denmark). Maximum likelihood analyses were performed with the software PhyML 3.0 [16]. Nucleotide substitution models were selected with Model Generator 0.85 [17] and nodal supports assessed with 1000 bootstrap replicates. Nucleotide sequences generated in this study were deposited in Genbank under accession numbers KF725664 to KF725686.

Blood films were collected for the 30 frigatebirds we sampled in 2012 on Europa (Table 1). These films were fixed in absolute methanol in the field, stained with Giemsa back in the laboratory, and examined at high magnification (x1000) in order to assess the number of parasites per at least 10000 red blood cells [5], [18]. A Euromex Oxion microscope (Euromex Microscopen BV, Arnhem, The Netherlands) was used to examine slides.

Statistical analyses were performed with the R.3.0.0 [19] software in order to compare infection rates between chicks and adults (Chi-squared test) and to detect a potential relationship between age of chicks (based on flattened wing measurements) and their parasitic status (Student’s t-test).

## Results and Discussion

Of the 209 collected blood samples (Table 1), 21 tested positive for the presence of *Haemoproteus* or *Plasmodium* parasites, in frigatebirds only. Based on partial Cytochrome *b* gene sequencing, 17 samples were confirmed to be positive for *Haemoproteus* and one for *Plasmodium*. Overall, 35% (±95% confidence interval: 13%) of tested great frigatebirds were thus positive for the presence of *Haemoproteus* (9 positive samples in 2011 and 8 positive samples in 2012). The three remaining PCR positive samples could not be sequenced because of limited DNA quantity. One of the 41 hippoboscid flies tested positive for the presence of *Haemoproteus.* Although based on a limited sample size, the estimated prevalence of great frigatebirds infected with *Haemoproteus* parasites was comparable to other studies on frigatebirds, usually ranging from 30% to 56% [2]. No difference was observed between adults (36%±18%) and chicks (35%±21%) (chi-squared test, P>0.05). Additionally, no relationship was detected between the age of the chicks and the infection status (Student’s t-test, P>0.05). Blood films examinations did not led to the observation of parasites of any blood stages, suggesting an extremely light parasite load.

**Figure 1:**
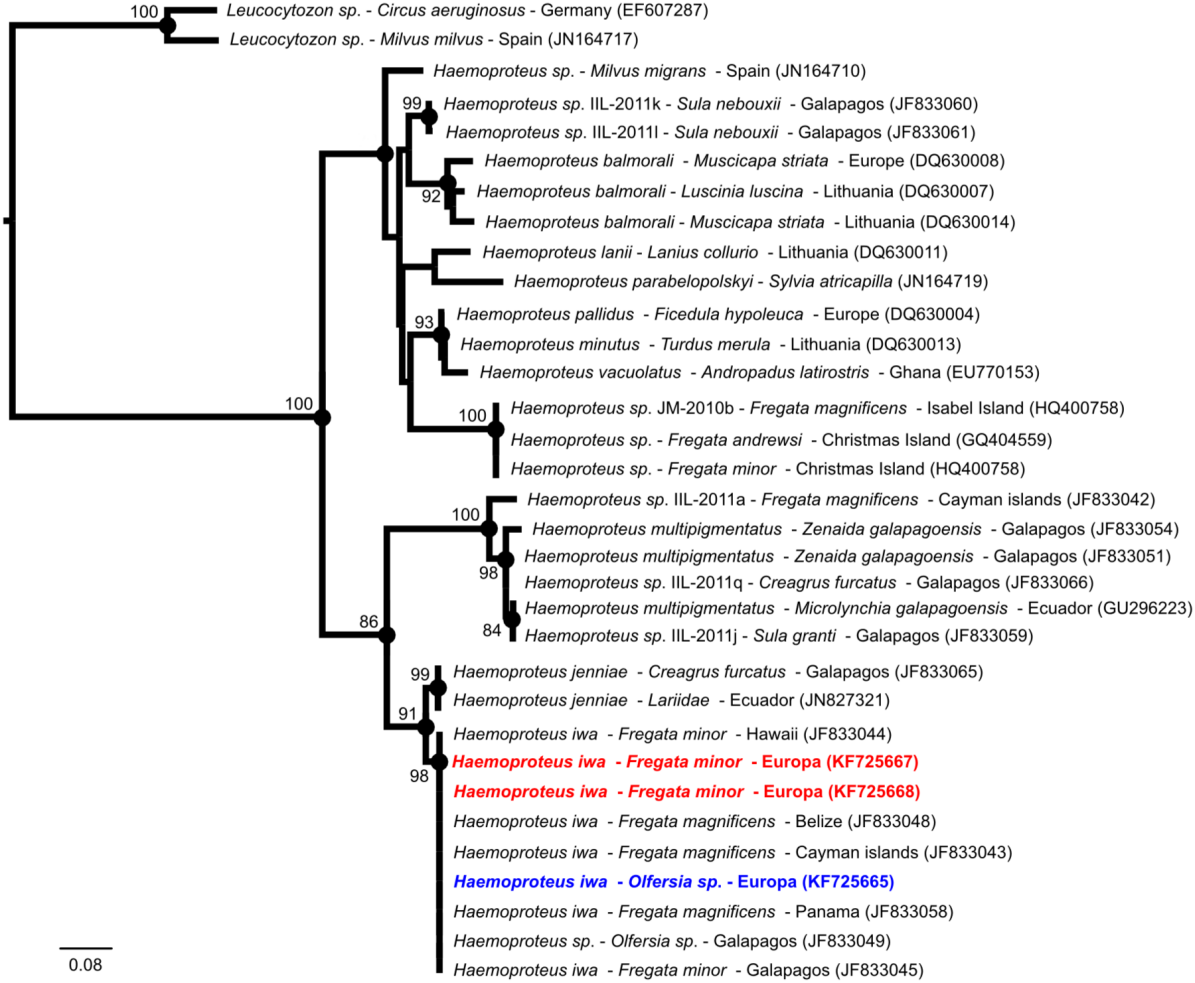
Maximum-likelihood consensus tree derived from 33 mitochondrial Cytochrome *b* nucleotide sequences (478 bp). Computations were performed with the general time reversible (GTR) nucleotide substitution model, an estimation of the proportion of invariable sites (I = 0.52) and of the nucleotide heterogeneity of substitution rates (α = 1.31). *Haemoproteus* parasite species, host species and geographic origin are indicated, when available. Two *Haemoproteus iwa* sequences derived from this study were included and are indicated in red; the sequence obtained from a *Haemoproteus* parasite detected in a hippoboscid fly is colored in blue. Bootstrap values were reported when higher than 80. Nucleotide sequence accession numbers are indicated in parenthesis.

Genetic analyses support that the detected *Haemoproteus* parasite was closely related to *Haemoproteus iwa* previously detected in frigatebirds in the Pacific Ocean and in the Caribbean Sea (Figure 1), in Hawaii, the Pacific coast of Panama, the Cayman Islands and the Galapagos Islands [5], as well as Mexico [20] and Christmas Island [7]. *Haemoproteus* sequences derived from this study were 100% identical to each other and had a perfect match to *Haemoproteus iwa* detected in frigatebirds in other geographic locations; however, as this was based on a limited alignment (478 bp), we cannot determine whether or not the apparent low genetic diversity of this parasite resulted from the limited length of nucleotide sequences available in public database. Genotyping of other genes such as the caseinolytic protease C encoding gene [4]–[5] could provide conformation on the putative parasite species and also reveal the potential genetic diversity of *Haemoproteus iwa* on Europa and among distant bird populations, worldwide.

Our findings support a large geographic distribution of *Haemoproteus iwa* associated with frigatebird breeding sites. Long-distance migrations may favor *Haemoproteus* parasites exchanges between breeding colonies. Based on satellite telemetry, Weimerskirch et al. [21] showed that a great frigatebird moved 4400 km from Europa to their roosting sites in Maldive Islands. More recent tracking studies conducted on great and lesser frigatebirds (*Fregata ariel*) from Europa have confirmed that both species disperse widely after breeding. One Lesser frigatebird tracked for about four months during the postbreeding migration crossed the entire tropical Indian Ocean from Europa to Indonesia, reached the Indonesian Sea via the Sunda strait and continued flying East up to the Salomon Island (Le Corre et al. in preparation), suggesting that frigatebird populations of the Pacific and Indian Ocean are interconnected. Combined with the study of frigatebird migrations, population genetic studies may provide new insight on parasite dispersal between oceanic islands.

Although the absence of *Haemoproteus* in boobies has been described before [5], [22]–[23], the lack of positive detection in red-footed boobies on Europa is particularly surprising given the geographic proximity and interactions with frigatebirds. Red-footed boobies are nesting inside the frigatebirds colony, sometimes on the same tree (Matthieu Bastien, personal observation). In spite of this mixing with frigatebirds nesting sites, no hippoboscid flies were found on boobies, suggesting a strict host-vector specificity which could in turn explain the absence of *Haemoproteus* infection in red-footed boobies on Europa.

**Figure 2:**
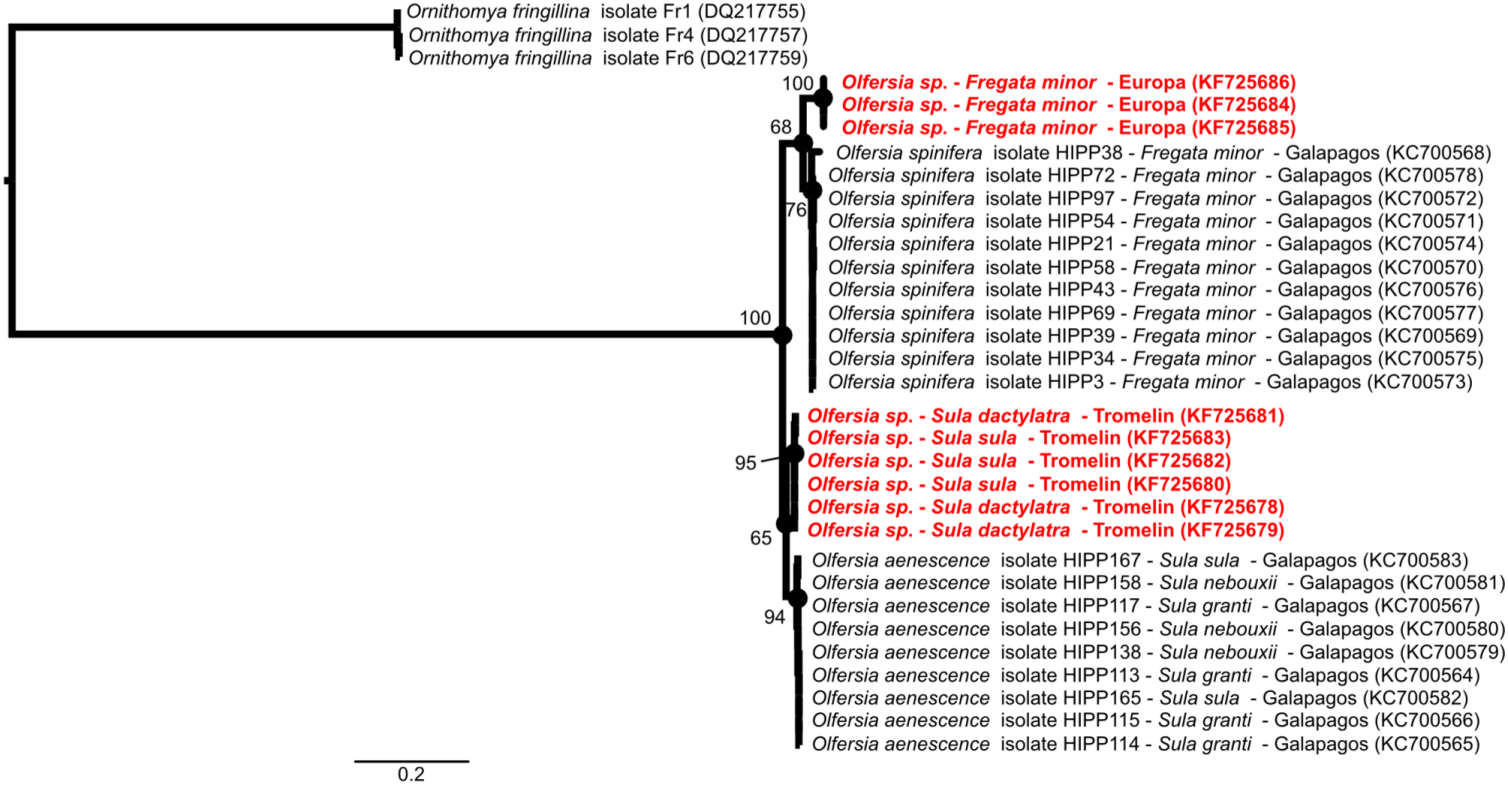
Maximum-likelihood consensus tree derived from 32 mitochondrial Cytochrome oxydase I nucleotide sequences (698 bp). Computations were performed with the unequal-frequency Kimura 3-parameter (K81 uf) nucleotide substitution model and an estimation of the nucleotide heterogeneity of substitution rates (α = 1.64). Hippoboscid fly species, host species and geographic origin are indicated. Sequences derived from this study are indicated in red. Nucleotide sequence accession numbers are indicated in parenthesis.

Genetic analyses also revealed that hippoboscid flies sampled from great frigatebirds on Europa were closely related to *Olfersia spinifera* in the Galapagos Islands (Figure 2). Flies collected on Tromelin on the two booby species were genetically related to *Olfersia aenescens* sampled on blue-footed boobies (*Sula nebouxii*). This finding further supports some level of specificity between the hippoboscid flies and their vertebrate hosts, as recently suggested by Levin and Parker [6], [11]. Although masked and red-footed boobies on Tromelin were heavily infested with hippoboscid flies (Matthieu Bastien, personal observation), none of the collected samples tested positive for the presence of *Haemoproteus*, supporting that birds and/or hippoboscid flies (*Olfersia aenescens* related species) on Tromelin may not be competent for the transmission of *Haemoproteus iwa* or alternatively that the parasite is not prevalent on Tromelin. In contrast, one of the eleven Hippoboscid flies sampled on frigatebirds on Europa tested positive for *Haemoproteus iwa* (Figure 1), supporting that this hippoboscid fly species is actually involved in parasite transmission.

**Figure 3:**
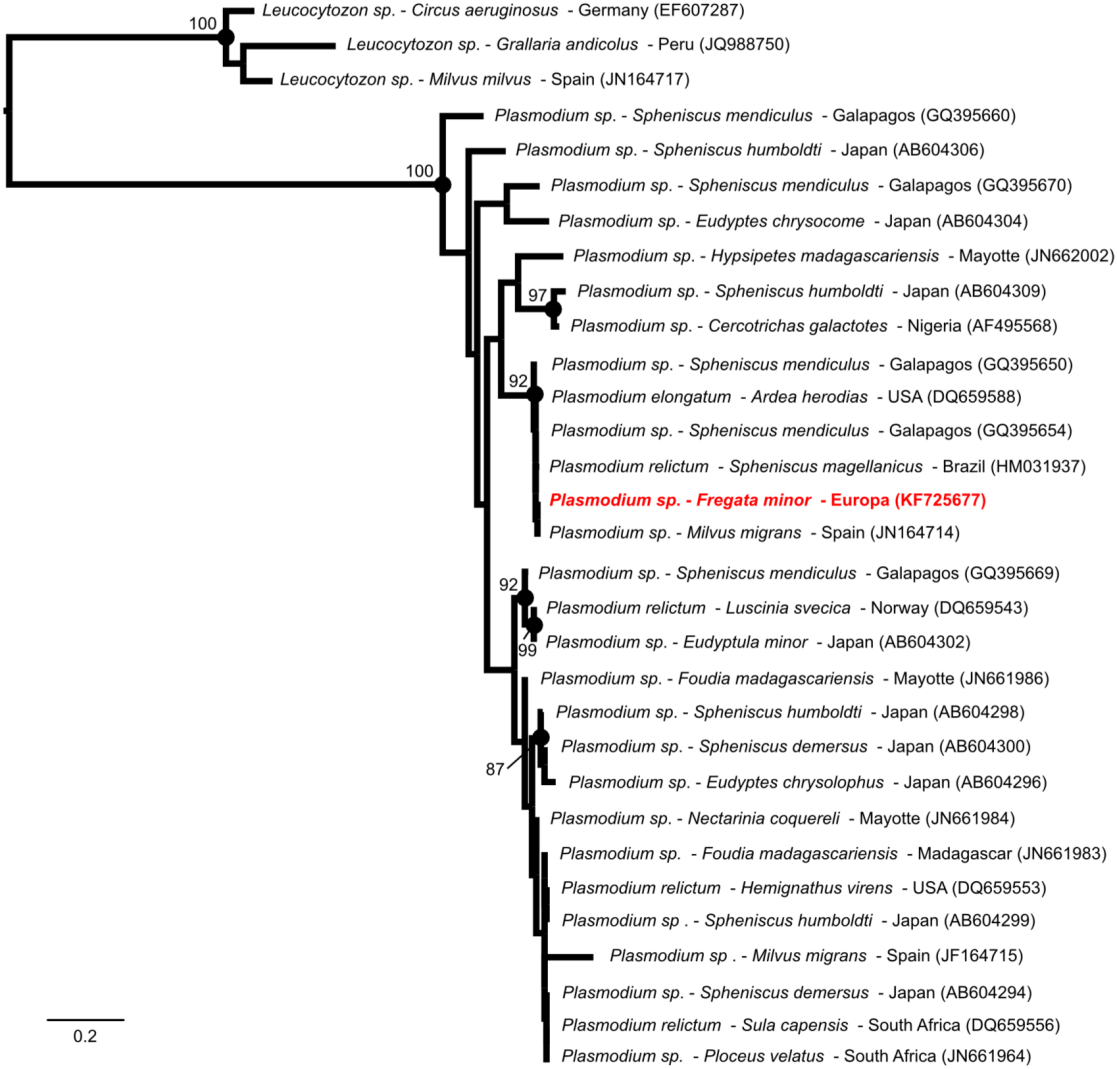
Maximum-likelihood consensus tree derived from 31 mitochondrial Cytochrome *b* nucleotide sequences (479 bp). Computations were performed with the general time reversible (GTR) nucleotide substitution model, an estimation of the proportion of invariable sites (I = 0.48) and of the nucleotide heterogeneity of substitution rates (α = 0.52). *Plasmodium* parasite species, host species and geographic origin are indicated, when available. The *Plasmodium* sequence derived from this study is indicated in red. Bootstrap values were reported when higher than 80. Nucleotide sequence accession numbers are indicated in parenthesis.

Our genetic analyses also supported that the *Plasmodium* we detected in a great frigatebird on Europa was genetically related to *Plasmodium* species identified in a large range of hosts (herons, penguins, kites) and geographic locations (Figure 3). Although the exact placement of the *Plasmodium* on the phylogenetic tree could not be resolved, this result nevertheless suggests that haemosporidian parasites other than *Haemoproteus* may be transmitted in seabird populations in the Western Indian Ocean. *Plasmodium* detection in seabirds is uncommon [2], potentially because of the absence of competent vectors in marine and coastal environments [3]. On Europa, however, the presence of a mangrove habitat favors the maintenance of notoriously dense populations of several mosquitoes species, including *Culex sitiens* [24], vector of *Plasmodium juxtanucleare* [25]. The peculiar host and vector species diversity found on Europa could favor parasite transmission as compared to oceanic islands such as Tromelin that are characterized by limited species richness and abundance. Future investigations are needed to determine if *Plasmodium* is actually transmitted by mosquitoes on Europa or if frigatebirds could be infected elsewhere during their foraging trip, or before their arrival on Europa for breeding. The complete host and geographic distribution of this parasite in the islands of the Western Indian Ocean also remains to be identified.
